# Chronic critical illness: are we saving patients or creating victims?

**DOI:** 10.5935/0103-507X.20170013

**Published:** 2017

**Authors:** Sergio Henrique Loss, Diego Silva Leite Nunes, Oellen Stuani Franzosi, Gabriela Soranço Salazar, Cassiano Teixeira, Silvia Regina Rios Vieira

**Affiliations:** 1 Programa de Pós-graduação em Ciências Médicas, Faculdade de Medicina, Universidade Federal do Rio Grande do Sul - Porto Alegre (RS), Brasil.; 2 Unidade de Terapia Intensiva, Hospital de Clínicas de Porto Alegre - Porto Alegre (RS), Brasil.; 3 Departamento de Nutrição, Hospital de Clínicas de Porto Alegre - Porto Alegre (RS), Brasil.; 4 Departamento de Nutrição, Hospital Mãe de Deus - Porto Alegre (RS), Brasil.; 5 Faculdade de Medicina, Universidade Federal de Ciências da Saúde de Porto Alegre - Porto Alegre (RS), Brasil.; 6 Departamento de Clínica Médica, Universidade Federal do Rio Grande do Sul - Porto Alegre (RS), Brasil.

**Keywords:** Critical illness, Respiration, artificial, Tracheotomy, Chronic disease, Allostasis, Mortality

## Abstract

The technological advancements that allow support for organ dysfunction have led to an increase in survival rates for the most critically ill patients. Some of these patients survive the initial acute critical condition but continue to suffer from organ dysfunction and remain in an inflammatory state for long periods of time. This group of critically ill patients has been described since the 1980s and has had different diagnostic criteria over the years. These patients are known to have lengthy hospital stays, undergo significant alterations in muscle and bone metabolism, show immunodeficiency, consume substantial health resources, have reduced functional and cognitive capacity after discharge, create a sizable workload for caregivers, and present high long-term mortality rates. The aim of this review is to report on the most current evidence in terms of the definition, pathophysiology, clinical manifestations, treatment, and prognosis of persistent critical illness.

## Abstract (pt)

Os avanços tecnológicos que permitem dar suporte às disfunções de órgãos levaram a um aumento nas taxas de sobrevivência para a maioria dos pacientes críticos. Alguns destes pacientes sobrevivem à condição crítica inicial, porém continuam a sofrer com disfunções de órgãos e permanecem em estado inflamatório por longos períodos. Este grupo de pacientes críticos foi descrito desde os anos 1980 e teve diferentes critérios diagnósticos ao longo dos anos. Sabe-se que estes pacientes têm longas permanências no hospital, sofrem importantes alterações do metabolismo muscular e ósseo, apresentam imunodeficiência, consomem quantias substanciais de recursos de saúde, têm reduzida capacidade funcional e cognitiva após a alta, demandam uma considerável carga de trabalho para seus cuidadores, e apresentam elevadas taxas de mortalidade em longo prazo. O objetivo desta revisão foi apresentar as evidências atuais, em termos de definição, fisiopatologia, manifestações clínicas, tratamento e prognóstico da doença crítica persistente.

## INTRODUCTION

Critically ill patients need intensive care since they are highly complex patients, requiring an active and multidisciplinary professional team as well as the use of advanced technology.^(1)^ The increased complexity of surgical procedures and other therapies provide a wider range of possibilities for the care of these patients than the care that existed in the first decades of intensive care units (ICUs). At that time, the most serious patients and those most refractory to therapeutic resources did not survive for long periods time.^(2)^

Advances in treatment approaches for critically ill patients, such as mechanical ventilation (MV), invasive and noninvasive monitoring, extracorporeal ventilation, and renal replacement therapy, along with a better understanding of pathophysiological behavior in critically ill patients, have led to reduced mortality rates in recent decades.^(3)^ However, although a few extremely severe patients survive for longer periods of hospitalization, there is nonetheless no significant decrease in the mortality rate of these patients.^(4-8)^ Moreover, those who do survive often develop permanent disabilities and experience intense suffering that can impact their entire families, changing their usual dynamics.^(9,10)^

Chronic critical illness (CCI) is characterized by lengthy hospital stays, intense suffering, high mortality rates and substantial resource consumption.^(11)^ Although CCI has been described for more than 40 years, we still know very little about the characteristics of this population, such as their risk factors, long-term mortality, functional capacity, cognition, and return to daily activities after hospital discharge. To further complicate the situation, the results of clinical trials vary from center to center.^(4,12,13)^ The time has come for us to give serious thought to this scenario. We should seek out alternatives to avoid an increase in CCI, develop protocols and strategies to improve patient recovery, and rethink how the resources available for critically ill patients are managed.

### Defining chronic critical illness

Patients with chronic critical illness typically have prolonged dependence on some form of life support.^(4)^ The prevalence of this syndrome ranges from 5 to 20% of patients admitted to the ICU.^(11)^ This wide variation can be explained by the lack of consensus on diagnostic criteria. Table 1 was extracted and modified from a 2010 review of diagnostic criteria for this syndrome.^(4,9,11,12,14-24)^ CCI patients are frequently dependent on prolonged ventilation support, and a period of three weeks or more on MV or the need for tracheotomy due to prolonged MV (PMV) were initially adopted as a consensus definition for the condition.^(22,25,26)^

**Table 1:** Time-related definitions and other features in chronic critical illness

Year	Author	Definition
2015	Kahn et al.^(14)^	8 or more days in an ICU with one or more of the following six conditions: MV, tracheotomy, stroke, head trauma, sepsis and serious injury
2013	Loss et al.^(11)^	21 days on MV or tracheotomy
2012	Carson et al.^(16)^	21 days on MV for at least six hours/day
2011	Boniatti et al.^(17)^	21 days on MV or tracheotomy
2008	Zilberberg et al.^(18)^	96 hours or more on MV
2007	Scheinhorn et al.^(15)^	Prolonged MV due to respiratory failure
2005	MacIntyre et al.^(19)^	21 days on MV for at least six hours/day
2005	Daly et al.^(20)^	72 hours or more on MV
2004	Nelson et al.^(9)^	Prolonged dependence on ventilatory support or tracheotomy associated with metabolic, neuroendocrine, neuropsychiatric and immunological changes
2002	Nierman^(21)^	Previous critical illness survival that presents significant functional impairment and dependence on intensive nursing care and advanced technology
2002	Carson e Bach^(22)^	21 or more days of continuous care and dependence on MV in an ICU
2000	Nasraway et al.^(23)^	Presence of severe previous diseases or complications during the ICU stay, often dependent on MV or renal replacement therapy
1997	Douglas et al.^(24)^	Required intensive nursing care and a length of stay of two weeks or more in an ICU
1985	Girard and Raffin^(4)^	No survival despite extraordinary life support for weeks to months

Adapted from: Wiencek C, Winkelman C. Chronic critical illness: prevalence, profile, and pathophysiology. AACN Adv Crit Care. 2010;21(1):44-61; quiz 63.^(12)^ ICU - intensive care unit; MV - mechanical ventilation.

The length of ventilatory support has been the most important marker of the syndrome; however, different periods of mechanical ventilation have been proposed.^(22)^ A two-week time frame is as efficient as that of a three-week time frame in identifying this population, although shorter time frames (such as four and seven days) have also been proposed.^(17,22,27,28)^ Nelson et al. have proposed a period of ten days of mechanical ventilation as indicating the appropriate moment for a tracheotomy and as a marker of the CCI period.^(29)^ CCI patients need to be distinguished from those who are dependent on mechanical ventilation as a result of respiratory and/or neuromuscular disorders and who do not meet the criteria for critical illness (or those who have overcome the critical illness and no longer present with the characteristics of the acute inflammatory phase). These patients are defined as dependent on prolonged ventilatory support.^(30)^

Patients with CCI are those who maintain a persistent inflammatory environment; humoral, hormonal and neuromuscular disorders with reduced immunity; and progressive consumption of physiological reserves.^(21,22,31-36)^ PICS, an acronym that means persistent inflammation, immunosuppression, and catabolism syndrome, has recently been used to define this scenario.^(35)^ In this context, CCI may be defined as an allostatic overload in more severe patients. Allostasis (allostatic load) comprises the organic modifications that ensure stability in adverse situations (food deprivation, inflammation, etc.) to support (new) homeostasis.

**Figure 1:**
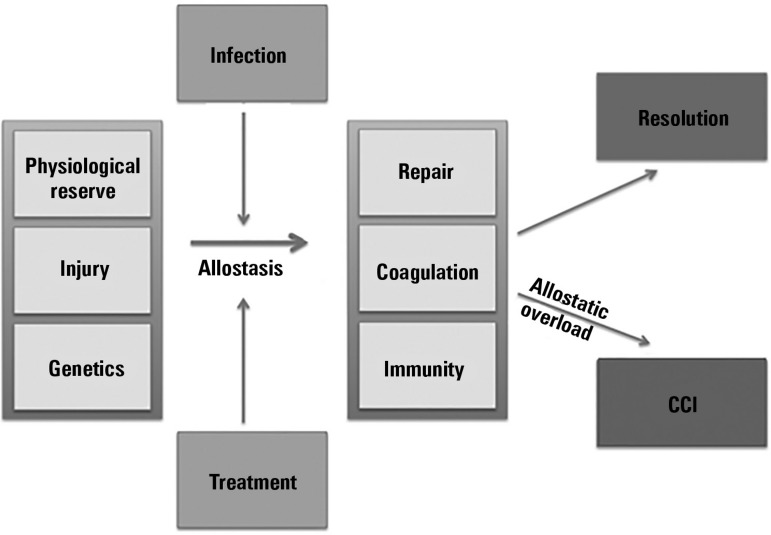
Injury, allostasis and allostatic overload. CCI - chronic critical illness.

Allostatic overload results from persistent insults and can be subdivided into type 1 (deprivation) and type 2 (excess). Type 1 allostatic overload can occur during extended periods of energy expenditure, exceeding actual energy consumption (e.g., deliberate and prolonged energy intake that does not meet the current demands of the patient or periods with no feeding and no adequate justification). Type 2 allostatic overload takes place when allostasis occurs in patients with persistent hyperglycemia, hypertriglyceridemia, hyperosmolarity, etc. (persistent inflammation and/or inadequate nutrition). Allostatic overload in severe critical patients can be a key element in the incidence of CCI^(37-39)^ (Figure 1).

Therefore, to better define different syndromes so that we can more homogeneously compare treatments and outcomes, new definitions have been proposed as follows: CCI, persistent critical illness (PCI), diseases that necessarily require long recovery periods, prolonged weaning from MV, and long ICU stay.^(30,40)^ Persistent critical illness is perhaps a more appropriate designation than CCI for the condition of prolonged life support, persistent low intensity inflammation, and multi-organ failure. Moreover, these abnormalities tend to be persistent or recurrent. We adopted CCI as a default designation in this review. It is very important that the reader pay attention to the different denominations when trying to contextualize the patient according to the best definition, i.e., CCI, PCI or PMV.

### Patients at risk for chronic critical illness

The prevalence of CCI has increased, and individuals suffering from chronic critical illness became complex and exhibit neurological, endocrine, metabolic, immunologic, and muscle disorders. There is no clear association between age and/or previous chronic disease and PCI, although once the transition from critically ill to a chronic condition is characterized the elderly tend to have higher rates of mortality.^(11)^

It is hard to characterize the transition to this different period of severe illness. However, the simultaneous association of a few variables, such as sepsis at the time of admission to the ICU, a need for invasive ventilatory support, mental changes, overweightness, and insufficient nutrition in the acute phase, were associated with chronicity 92% of the time in an observational cohort study conducted by our group.^(11)^ Variables used in other studies included age, previous chronic diseases, malnutrition, high severity scores at admission, need for enhanced invasive monitoring, and early development of organ dysfunction.^(16,41,42)^ A study conducted by Carson et al. validated a mortality prediction model for patients submitted to ventilatory support for twenty-one or more days. The study describes the need for vasopressors and/or hemodialysis, a platelet count below 150,000, and an older age (50 years old or older) as predictors of CCI.^(43)^ A large cohort of patients who received prolonged ventilatory support, totaling 817 critical patients, followed survivors one year after discharge. Worse results were observed in elderly patients, those with poor prognostic indicators, those with more comorbidities and those who depended on nursing care at admission. However, the criterion used to define PMV was 48 hours or more of ventilatory support.^(44)^ Clark and Lettieri studied the predictors for PMV at the time of intubation. Using the criterion of 14 days of ventilatory support, they noted that acidosis, renal failure, and tachycardia were very specific predictors.^(45)^

It is important to standardize definitions so that PMV and CCI can be understood and studied as separate entities, allowing for more appropriate prevention protocols and more specific therapies.

### Pathophysiology and recognition of chronic critical illness

Unlike critical illnesses with acute evolution, the persistence of the inflammatory environment in PCI patients induces changes in the hypothalamic-pituitary and adrenal axis in the form of changes in the serum levels of cortisol, renin, angiotensin, and aldosterone. This environment induces alterations in protein and bone metabolism, body composition, and vascular tone. As a result of these changes, there is fluid retention, skin vasoconstriction, and ulcerations. Muscle loss and edema cause weakness and dependence on ventilatory support.^(35,46)^

In experimental studies with rats undergoing MV for long periods of time, an analysis of the ultrastructure and mitochondrial activity of animal diaphragm myocytes shows cellular changes consistent with degeneration induced by hypoxia and oxidative stress.^(47-49)^ Strategies for ventilatory management and respiratory rehabilitation have been described for the treatment of patients undergoing prolonged periods of MV.^(50)^ Some muscle training strategies have shown promising results in small studies,^(51,52)^ as have strategies for long-term care after hospital discharge.^(20,53)^ However, metabolic intervention strategies for the factors associated with diaphragm muscle degeneration during PMV tested in rats have not been tested in humans.^(54)^

Patients with persistent critical illness are at risk of new infections during hospitalization because of the broken skin barrier (pressure sores, drains, and/or catheters), immunodeficiency due to progressive consumption of biological reserves, and sharing an environment inhabited by virulent microorganisms resistant to most antibiotics.^(8,55)^ CCI patients have alterations in hormone pulses (growth hormone and/or adrenal and thyroid hormones) and may even develop hypogonadism.^(34,41)^ Patients may also suffer from muscle atrophy (cachexia),^(31)^ insulin resistance, and hepatic steatosis, conditions resulting from this inflammatory environment.^(32,33,39)^ They are particularly vulnerable to parenteral nutrition-induced hyperglycemia and intravenous insulin-induced hypoglycemia.^(32,33,39)^ Most of these patients have pressure ulcers and receive multiple blood transfusions.^(11)^ Neuropsychiatric disorders are common, especially depression, memory loss, and changes in cognition.^(9,41,56,57)^ Among survivors, depression and reduced cognitive ability tend to persist after discharge.^(58)^

Chronic critical illness has no pathognomonic manifestations. Similar definitions for different contexts contribute to the confusion. Intensivists are not trained to consider CCI as a possible outcome for patients admitted to the ICU. A study conducted in Australia and New Zealand asked intensivists to identify which conditions, in their view, were associated with CCI. Every professional had to reply with at least one feature. The most common were respiratory failure, delirium, acquired muscle weakness, sepsis, renal failure, malnutrition, and pressure ulcers. The same study also asked what diseases had longer recovery periods, but without CCI. The most commonly cited diseases were neuromuscular disease, traumatic brain injury, and pancreatitis.^(40)^

### Economic impact of chronic critical illness

Chronic critical illness has substantial costs that sometimes amount to more than 60% of the total ICU cost.^(59)^ Although many cost assessment studies defined PMV patients as those submitted to MV for more than 96 hours (instead of fourteen or twenty-one days, the most current CCI definition), the fact that these patients stay for longer periods in the hospital, are readmitted more often and frequently have other disorders, such as renal failure requiring hemodialysis, results in a higher individual cost that is three to four times higher than the cost for critically ill patients who do not require PMV.^(59,60)^ One study showed that PMV patients (six months or more) significantly increased the average ICU cost.^(61)^ Another study demonstrated that CCI corresponded to 40% of the total ICU cost over six months.^(11)^ Deinstitutionalization strategies and home care can decrease costs, but are not always associated with better outcomes.^(62,63)^

### Treatment of chronic critical illness

There is no protocol or preferential approach to CCI. Perhaps the best approach at our disposal is organizing an appropriate early multidisciplinary therapy for severe critically ill patients after admission (or before ICU admission), aiming to reduce latency for antibiotics and nutrition, hemodynamic resuscitation, and gentle ventilation.^(11)^ Once the risk of CCI and PMV is observed, early tracheotomy (ten days of MV) should be considered.^(64,65)^ In PMV patients, spontaneous breathing through tracheotomy appears to be better than protocols using varying levels of pressure support over time in an attempt to wean them from ventilatory support.^(66)^

Nutrition is key for CCI patients. Patients should be fed, preferentially via the enteral route, to avoid inappropriately high or low caloric intake. Polymeric formulas should be tried first, with the use of semi-elemental formulas considered in cases of intestinal dysfunction. Indirect calorimetry is the gold standard to guide calorie intake, but this technology is not available in most ICUs. Predictive equations can be used to calculate energy expenditure, but one must keep in mind that these methods have not been validated and that the results are often inconsistent. Referencing the calorie intake to patient weight has been widely used, meaning that the recommended nutrient intake often ranges from 20 to 25kcal/kg/day, has high protein levels (> 1.2g/kg/day) and is high in vitamins and trace elements. Protein intake should not be restricted in patients undergoing renal replacement therapy. The need for protein, vitamins, and trace elements is greater in these patients due to loss through the capillary membrane. There is no recommended amount for these substrates. Hyperglycemia should be managed by adjusting carbohydrate intake (it may be necessary to reduce intake to less than 100g/day), the use of specific formulas for diabetes, and the administration of subcutaneous NPH insulin (and subcutaneous simple insulin given as a fixed dose or as a rescue dose). Intravenous insulin use should be the exception and must be avoided as much as possible. One-third of all patients suffer from diarrhea, which should be managed with the addition of soluble fiber (15 - 20g/day) and probiotics.^(34,39,67,68)^

Muscle dysfunction is one of the most easily noticeable dysfunctions in CCI patients and the one that regresses most slowly in survivors undergoing rehabilitation. More precisely, the condition is called ICU-acquired weakness (ICUAW), and it is a major marker of PMV. ICUAW results from inflammation, hyperglycemia, immobility, multi-organ dysfunction, and possibly some medications (steroids, sedatives, neuromuscular blockers). Patients suffer from proximal and symmetrical loss of muscle strength associated with changes in electromyography (widespread fibrillation and positive sharp waves, decreased amplitude of compound muscle and sensory nerve action potentials, and relatively normal conduction studies). Muscle biopsies reveal atrophy. There is no specific treatment, but efforts are focused on early mobility and judicious use of steroids, sedatives and analgesics as well as adequate glycemic control. Patients should be encouraged to get out of bed, even if receiving ventilatory support. Passive and active muscle rehabilitation strategies are important. Physical activity programs, including walking tests in the ICU, virtual reality games, exercise, and electro-stimulation, should be applied and monitored by specialized professionals. Protein supplements should be administered in conjunction with nutritional therapy.^(8,31,69,70)^

The administration of non-steroidal anabolic agents should be considered for patients with clear hypogonadism and/or severe cachexia, although this recommendation is not an evidence-based^(71)^ (of course, these patients should receive appropriate nutritional therapy and motor rehabilitation as well). Patients should be screened for osteopenia and osteoporosis using radiological techniques and clinical analysis (calcium, vitamin D, and parathyroid hormone). Hypovitaminosis D (less than 10pg/mL) and/or hyperparathyroidism indicate treatment with calcium and vitamin D.^(33,67)^ It should be stressed that bone resorption is also present with normal parathyroid hormone levels.^(72)^ These patients should also be screened and treated for hypophosphatemia and hypomagnesemia.^(33)^

The treatment of pressure ulcers is also important since they decrease the patient's self-esteem, hinder mobility and cause secondary infections. The staff should be on high alert for osteomyelitis in patients with deep bedsores and signs of systemic inflammation without an obvious site.^(73)^

There are no specific recommendations for blood cell transfusions in CCI patients. Hospital staff should follow the current guidelines for critically ill patients. This therapy is usually indicated by the clinical onset of anemic syndrome or very low hemoglobin levels (< 7 - 9g/dL). Patients repeatedly transfused are at a higher risk for complications of blood therapy (infection and acute lung injury).^(74,75)^

Finally, psychological support and the administration of antidepressants/antipsychotics are recommended for the management of depression or other mental changes. The medications used and their dosage vary widely and should be determined by a specialist, further reinforcing the concept of multidisciplinary treatment. The involvement of occupational therapists, physical educators, and social workers should be considered in all patients who are undergoing rehabilitation. Family training is a very important component and key to success in the rehabilitation period.

### Prognosis

Chronic critical illness is characterized by hospital admissions with longer lengths of stay, higher mortality rates and increased cost. Our 2013 observational cohort study showed a mortality rate of 32% at the ICU, while in the hospital as a whole it reached 56%.^(11)^ Hospital mortality was even higher (65%) in our multicenter cohort in 2015.^(26)^ Other cohorts have produced similar data.^(17,42,76)^ Among survivors discharged from the hospital, results do not change significantly. Mortality from six to 12 months after discharge ranged from 40 to 67%,^(16,61,76-78)^ and was even higher (74%) for patients who were discharged from the hospital but needed some form of ventilatory support at home.^(61)^ Patients over 75 years old, or over 65 years old who also had impaired functional capacity, had a mortality rate of 95% after one year in a study by Carson et al.^(79)^ Hartl et al.^(78)^ followed CCI patients discharged from the hospital for up five years and found an 80% mortality rate during this period. The multicenter study by Combes et al. included 17 ICUs, assessing functional capacity for a population of 141 chronic critical patients 57 months after discharge. They found that these patients had significantly lower functional capacity compared to the general population from the same location.^(80)^

These data reveal the extreme seriousness and fragility of CCI patients and lead us to wonder whether our ICUs generate survivors or victims of critical illness and its treatment, especially considering their low survival rates during a relatively short period after discharge (one year) as well as concomitant limitations and significant suffering.

### Strategies to reduce the incidence and prevalence of chronic critical illness and rehabilitation

Contemporary critical care specialists should certainly be familiar with the technologies and challenges of modern intensive care. However, they should also be alert to the unexpected results of these therapeutic processes, such as CCI. Patients who develop this syndrome have a poor prognosis and experience intense suffering, forcing us to wonder whether we are actually making them victims of intensive care.

To decrease the incidence of CCI, the best medical practice immediately after admission to the ICU might be the proper use of bundles of treatment and following the prudent recommendation that "less is more"^(81)^ (less aggressive ventilatory support, lower calorie intake, less fluid administration, lower doses and shorter sedation times). A Cochrane systematic review and meta-analysis showed that the adoption of a validated protocol in patients undergoing mechanical ventilation reduced the MV period, weaning period, and ICU stay.^(82)^

Once CCI is detected, the adoption of a proper treatment plan for this phase of the evolution of critically ill patients, associated with the challenges of discovering new treatments for them, may contribute to shortening this period and give the patients a chance to fully recover so that they may return to their previous functional status. New therapies must cover the restoration of proper body composition and a full functional recovery.

## CONCLUSION

Critically ill patients are at risk for chronic critical illness, a syndrome characterized primarily by longer hospital stays, high costs, reduced hospital and post-hospital survival and intense suffering. A set of therapies focused on restoring mobility, body composition, and function has been proposed, but the prevalence of chronic critical illness remains high, generating elevated costs and significant restrictions for survivors. We should redouble our efforts to learn more about the syndrome in an attempt to decrease its incidence and improve outcomes.
